# Pattern of self-reported adverse events related to COVID-19 vaccines in Saudi Arabia: A nationwide study

**DOI:** 10.3389/fpubh.2023.1043696

**Published:** 2023-02-23

**Authors:** Joud Mohammed Alkhalifah, Ahad Al Seraihi, Jaffar A. Al-Tawfiq, Badr Fadhel Alshehri, Alhanouf Hani Alhaluli, Naif Mansour Alsulais, Mohammed Mesfer Alessa, Waleed Seddiq, Thamer Aljeri, Mohammad Hassan Qahtani, Mazin Barry, Maram Al-Otaiby

**Affiliations:** ^1^College of Medicine, King Saud University, Riyadh, Saudi Arabia; ^2^The Saudi Ministry of Health, Riyadh, Saudi Arabia; ^3^Infectious Disease Unit, Specialty Internal Medicine, Johns Hopkins Aramco Healthcare, Dhahran, Saudi Arabia; ^4^Infectious Disease Division, Department of Medicine, Indiana University School of Medicine, Indianapolis, IN, United States; ^5^Infectious Disease Division, Department of Medicine, Johns Hopkins University School of Medicine, Baltimore, MD, United States; ^6^Center for Stem Cell and Translational Immunotherapy (CSTI), Harvard Medical School, Boston, MA, United States; ^7^Department of Neurosurgery, Brigham and Women's Hospital, Harvard Medical School, Boston, MA, United States; ^8^Division of Infectious Diseases, Department of Internal Medicine, College of Medicine, King Saud University, Riyadh, Saudi Arabia; ^9^Division of Infectious Diseases, Faculty of Medicine, University of Ottawa, Ottawa, ON, Canada; ^10^Department of Pathology, College of Medicine, King Saud University, Riyadh, Saudi Arabia

**Keywords:** COVID-19, vaccines, side-effects, adverse events, self-reported, cohort, registry, Saudi Arabia

## Abstract

**Background:**

Vaccination against coronavirus disease 2019 (COVID-19) is the most effective way to end the pandemic. Any development of adverse events (AEs) from various vaccines should be reported. We therefore aimed to explore major and minor AEs among vaccinated individuals in Saudi Arabia.

**Methods:**

This is a nationwide report based on the Saudi Arabian Ministry of Health (MOH) registry. It included those who received COVID-19 vaccines from 17th December 2020 to 31st December 2021. The study included spontaneous self-reported adverse effects to COVID-19 vaccines where the study participants used a governmental mobile app (Sehhaty) to report their AEs following vaccination using a checklist option that included a selection of side-effects. The primary outcome was to determine AEs reported within 14 days of vaccination which included injection site itching, pain, reaction, redness, swelling, anxiety, dizziness, fever, headache, hoarseness, itchiness, loss of consciousness, nausea, heartburn, sleep disruption, fatigue, seizures, anaphylaxis, shortness of breath, wheezing, swelling of lips, face, and throat, loss of consciousness, and admissions into the intensive care unit (ICU).

**Results:**

The study included a total number of 28,031 individuals who reported 71,480 adverse events (AEs); which were further classified into minor and major adverse events including ICU admissions post vaccination. Of the reported AEs, 38,309 (53. 6%) side-effects were reported following Pfizer-BioNTech, 32,223 (45%) following Oxford-AstraZeneca, and 948 (1.3%) following Moderna. The following reported AEs were statistically significant between the different vaccine types: shortness of breath\difficulty of breathing, dizziness, fever above 39°C, headache, hoarseness, injection site reactions, itchiness, nausea, sleep disruption, fatigue, wheezing, swelling of lips/face and\or throat, and loss of consciousness (*p*-value < 0.05). Fever and seizure were the only statistically significant AEs amongst the number of vaccine doses received (*p*-value < 0.05). Ten ICU admissions were reported in the 14 days observation period post-COVID-19 vaccination with the following diagnoses: acute myocardial infarction, pneumonia, atherosclerosis, acute respiratory failure, intracranial hemorrhage, grand mal seizure, Guillain-Barré syndrome, abnormal blood gas levels, and septic shock.

**Conclusion:**

This study demonstrated that the most prevalent SARS-CoV-2 vaccine side-effects among adults in Saudi Arabia were mild in nature. This information will help reduce vaccine hesitancy and encourage further mass vaccination to combat the COVID-19 pandemic, especially as booster doses are now available. Further studies are warranted to obtain a better understanding of the association between risk factors and the experiencing of side-effects post vaccination.

## 1. Introduction

The coronavirus disease (COVID-19) pandemic has caused a serious threat and catastrophes worldwide. As of May 20, 2022, the virus had caused more than 521 million confirmed cases, with 6 million deaths worldwide as reported by the World Health Organization (WHO) (1). In Saudi Arabia, however, COVID-19 has infected 762,575 people and resulted in 9,128 deaths, according to the Saudi Ministry of Health (MOH) COVID-19 dashboards.

To combat the pandemic, multiple vaccines have been developed. The most used vaccines include Pfizer-BioNTech (BNT162b2), Moderna (mRNA-1273), and Oxford–AstraZeneca (ChAdOx1). As of May 20, 2022, more than 11 billion vaccine doses have been administered globally (1). Post-vaccination adverse events (AEs) reporting and safety monitoring are recommended by multiple institutions worldwide, especially during a pandemic (2).

The Kingdom of Saudi Arabia (KSA) has implemented multiple strategies to mitigate the impact of COVID-19 pandemic and was indeed one of the first countries to launch a nationwide mass vaccination campaign against COVID-19 (3–5). Indeed, The Saudi Food and Drug Authority (SFDA) granted emergency use authorization for the use of Pfizer-BioNTech COVID-19 vaccine on the same day the Phase III trial results were published (6, 7). Six days later, on December 17, 2020, Saudi Arabia received its first shipments of BNT162b2 and immediately started the mass vaccination campaign based on a phased approach to vaccinate the adult citizens and residents free of charge (3). Subsequently on February 19, 2021, Saudi Arabia authorized the use of AstraZeneca-ChAdOx1 vaccine for emergency use, which was then followed by the approval of Moderna-mRNA-1273 vaccine on July 09, 2021. The vaccine distribution was based on high-risk categories identified by Saudi MOH, as follows; the first phase targeted high risk groups which included citizens over 65 years old, professionals who are most vulnerable to infection, obese people with a BMI >40 kg/m2, immunocompromised citizens and citizens with two or more of the following chronic conditions: asthma, diabetes, chronic kidney disease, chronic heart disease, including coronary artery disease, and chronic obstructive pulmonary disease, and those with a previous stroke. The second phase targeted citizens over 50 years old, and those with one of the chronic illnesses mentioned above. The third phase included all citizens and residents 16 years or older that wish to be vaccinated (8). The estimated period for vaccination completion was July 2021 for phase one and two, and September 2021 for phase three (5). On June 27, 2021, the SFDA approved Pfizer-BioNTech COVID-19 vaccine for Saudi citizens and residents between 12 and 18 years old, and the Saudi MOH started campaigns to encourage adolescents to register for vaccination (9).

In order to combat the SARS-CoV-2 pandemic, the Kingdom has developed their digital health system through multiple applications that target different facets of managing this ongoing pandemic, one of these applications named “Sehhaty” was created to manage to aid users in managing their COVID-19 related services, including booking vaccination appointments, COVID-19 testing appointments, viewing testing results, reporting any side-effects and many other services (10). The WHO reported that as of May 14, 2022, more than 64 million vaccine doses were administered in KSA (1).

Vaccine hesitancy is one of the main barriers of vaccine uptake. One study from KSA showed that one-third of healthcare workers (HCWs) received the COVID-19 vaccine during the first month of launching the vaccine campaign (11). Another study of 23,000 surveyed HCWs in KSA showed that the most common reason for vaccine hesitancy was fear of potential AE as reported by 60% of respondents (12). Few studies reported such occurrences of AEs following COVID-19 in KSA and these studies were limited to single centers and to specific types of vaccines (13–15). However, this study represented the first national report of vaccine related AEs across the Kingdom using a national database in relation to the different vaccine types and different number of vaccine doses.

## 2. Materials and methods

### 2.1. Study design and settings

This is a retrospective, population-based, observational study examining the patterns of AEs secondary to COVID-19 vaccines as reported by vaccine recipients over the period from 17th December 2020 to 31st December 2021 to the Saudi MOH. The self-reported data of adverse vaccine side-effects were obtained from the Sehhaty mobile application and integrated with the national vaccination registry (NVR), and the health electronic surveillance network (HESN) databases. Furthermore, the ICU patient data were obtained from a national database called Ehalat and were further integrated with the aforementioned databases. Data privacy and confidentiality were maintained throughout the study. Informed consent was waived due to the retrospective and observational nature of the study.

### 2.2. Data collection

#### 2.2.1. Sehhaty application

The Sehhaty application was launched under the umbrella of digital transformation initiatives by the Saudi MOH aiming to provide access to several healthcare services for individuals in the Kingdom. These services included consultations, PCR testing for SARS-CoV-2, booking COVID-19 vaccine appointments, and reporting COVID-19 vaccines side-effects through the option of manual entering and/or drop box option selection (16). Indeed, the Sehhaty application offers a checkbox option to report symptoms for the received vaccine. The following symptoms were available for users to select from in the following order: injection site itching, wheezing, fatigue, hoarseness, low fever, anxiety, fever above or below 39°C, heartburn, injection site swelling, headache, loss of consciousness, nausea, injection site redness, injection site pain, itchiness other than injection site, seizure, dizziness, difficulty or shortness of breath, sleep disruption, swelling of lips, face, and throat, and intensive care unit (ICU) admission. The study participants were provided with a checkbox list that included all of the aforementioned side-effects and were given the option to select from the list if they experienced any of them. Another helpful feature was the registration of the date and the start of symptoms, which allowed for filtering and limiting the studied side-effects to be within a window of 14 days post-vaccination (7). Participants' demographics data were collected and included: age, gender, nationality, region, COVID-19 vaccine type, the number of vaccine doses, date of vaccination, and date of ICU admission.

### 2.3. Study participants

The study included all vaccinated female and male individuals in Saudi Arabia of any ethnicity, who were 12–96 years of age and spontaneously self-reported their side-effects through the Sehhaty app after receiving at least one dose of the three available COVID-19 vaccines: Pfizer–BioNTech (BNT162b2), Oxford–AstraZeneca (ChAdOx1-S), and/or Moderna (mRNA-1273) within 14 days of receiving COVID-19 vaccination over the period from 17th December 2020 to 31st December 2021 (7). The ICU records were considered post vaccine events if they happened within 14 days post COVID-19 vaccination. The exclusion criteria entailed individuals with wrong or missing data entry and/or misidentified unique IDs, individuals who are under 12 years or above 96 years old, those who did not receive COVID-19 vaccination, or who received COVID-19 vaccines other than Pfizer–BioNTech, Oxford–AstraZeneca or Moderna vaccines, and those who reported their side-effects outside of the 14-day window. We excluded those with pre-existing comorbidities and/or autoimmune diseases, as they could confound the results of the self-reported vaccines adverse events. The primary objective was to determine the patterns of minor side-effects: injection site (itchiness, pain, reaction, redness, swelling, and other), anxiety, dizziness, fever above or below 39°C, headache, hoarseness, itchiness other than injection site, loss of consciousness, nausea, heartburn, sleep disruption, and fatigue. In addition, we examined the pattern of reported major COVID-19 vaccine side-effects: seizures, shortness of breath, wheezing, swelling of lips, face, and throat, ICU admission, and loss of consciousness. The AEs were classified as local, systemic, or allergic, or as mild or moderate.

### 2.4. Statistical analysis

Data were analyzed using the Statistical Package for the Social Sciences (SPSS) version 26.0 statistical software tool (IBM Statistics, Armnok, NJ, USA). Data were represented using descriptive statistics including (frequency, percentage tables, means, and standard deviations (SD), accordingly. The Chi-square test was used to determine statistically significant differences between vaccine types and number of doses, and the major and minor vaccine side-effects. Logistic regression analyses with the determinants of *p*-value and 95% confidence intervals (CIs) were then performed to compare demographic variables with the pattern of vaccine minor and/or major adverse events. A *p*-value of < 0.05 was used to report the statistical significance.

### 2.5. Ethical considerations

This study was ethically approved by the Institutional Review Board (IRB) committee from the General Directorate for Research and Studies (GDRS) (IRB No. 21-50M) at the Saudi MOH, KSA, with a waiver of the need for informed consent.

## 3. Results

### 3.1. Demographic characteristics of COVID-19 vaccine recipients reporting AEs

The total number of COVID-19 vaccine recipients who spontaneously reported any AE was 28,031 between 17 December 2020 and 31 December 2021 with an estimated reported incidence of 4.2 AEs per 10,000 doses. Table 1 represents the baseline demographic characteristics of the study participants in which the majority (*n* = 24,683; 88.1%) were Saudi nationals. A slightly higher proportion of the participants were females (54.3%; *n* = 15,231) compared with (45.7%; *n* = 12,800) males. Of the participants, the highest percentage of respondents was 8,791 (31.4%) from 30-39 years of age, whilst participants between 90 and 96 years and between 12 and 19 years were 20 (0.07%), and 1,043 (3.7%), respectively. The geographic distribution of the study participants varied between the 13 Saudi regions; 38.6% were from Riyadh followed by Makkah Al-Mukarramah (27.9%), and the Eastern Region (15.5%). Of the different COVID-19 vaccines, 15,111 (53.9%) received the Pfizer-BioNTech vaccine, and 12,601 (45%) received the Oxford–AstraZeneca vaccine, whilst only 319 (1.1%) received the Moderna vaccine. Of the included respondents, 21,126 (75.4%) received the first vaccine dose, 6,822 (24.3%) received the second dose, and only 83 (0.3%) reported receiving the third vaccine dose during the study period (Table 1).

**Table 1:** Demographic characteristics of COVID-19 vaccine recipients.

**Parameters**	**Frequency (percentage)**
**Age**	
12–19 yrs	1,043 (3.7%)
20–29 yrs	7,161 (25.5%)
30–39 yrs	8,791(31.4%)
40–49 yrs	5,059 (18%)
50–59 yrs	3,303 (11.8%)
60–69 yrs	2,011(7.2%)
70–79 yrs	496 (1.77%)
80–89 yrs	147 (0.5%)
90–96 yrs	20 (0.07%)
**Gender**	
Male	12,800 (45.7%)
Female	15,231 (54.3%)
**Nationality**	
Saudi	24,683 (88.1%)
Non-Saudi	3,348 (11.9%)
**Region**	
Al Baha	176 (0.6%)
Al Jawf	175 (0.6%)
Al Qassim	976 (3.5%)
Asir	952 (3.4%)
Hail	281 (1%)
Jazan	355 (1.3%)
Madinah	1,415 (5%)
Makkah Al Mukarramah	7,810 (27.9%)
Najran	195 (0.7%)
Northern borders	141 (0.5%)
Riyadh	10,826 (38.6%)
Eastern region	4,354 (15.5%)
Tabuk	375 (1.3)
**Vaccine type received**	
Moderna	319 (1.1%)
Oxford-AstraZeneca	12,601 (45%)
Pfizer-BioNTech	15,111 (53.9%)
**Vaccine dose received**	
1	21,126 (75.4%)
2	6,822 (24.3%)
3	83 (0.3%)
**Chronic diseases**	
Diabetes	5 (0.0178%)
Hypertension	6 (0.0214%)
Asthma	1 (0.0035%)
Coronary heart disease	1 (0.0035%)

### 3.2. Side-effects of COVID-19 vaccination by vaccine type

The study included a total number of 28,031 individuals who reported 71,480 AEs. Of the reported AEs, 38,309 (53.6%), 32,223 (45%), and 948 (1.3%) were after receiving the Pfizer-BioNTech, Oxford-AstraZeneca, and Moderna vaccines, respectively (Table 2). The most common reported AEs after Pfizer-BioNTech vaccine were fatigue 7,028 (18.35%), others 5,838 (15.24%), fever < 39°C 5,151 (13.45%), and headache 5,081 (13.26%). For Astra-Zeneca vaccine, the following were the most commonly reported AEs: fatigue 6,256 (19.41%), others 5,186 (16.09%), fever < 39°C 4,431 (13.75%), and injection site reactions 4,063 (12.61%). For the Moderna vaccine, however, the following were the most commonly reported AEs: others 200 (21.1%), fatigue 164 (17.3%), injection site reactions 119 (12.55%), and fever < 39°C 113 (11.92%). The following reported AEs were statistically significant between the different vaccine types received: shortness of breath\difficulty of breathing, dizziness, fever above 39°C headache, hoarseness, injection site reactions, itchiness other than injection site, nausea, sleep disruption, fatigue, wheezing, swelling of lips/face and\or throat, and loss of consciousness (*p*-value < 0.05). The rate of reported major AEs were as follows: 300 (0.42%), 270 (0.83%), and 13 (1.37%) after receiving the Pfizer-BioNTech, Oxford-AstraZeneca, and Moderna vaccines, respectively (Table 2). The rate of intensive care admission after vaccination was only 10 (0.03%) of all cases.

**Table 2:** Adverse events in relation to the type of vaccine received.

	**Pfizer-BioNTech**		**Oxford-AstraZeneca**		**Moderna**		**P-value**

	***n***	**%**	***n***	**%**	***n***	**%**	
**Minor side effects**							
Anxiety	221	0.58	207	0.64	5	0.53	0.48
Shortness of breath\difficulty of breathing	954	2.49	751	2.33	31	3.27	**0.015**
Dizziness	1,916	5.00	1,441	4.47	47	4.96	**0.002**
Fever above 39	3,180	8.30	2,284	7.09	52	5.49	**0.001**
Fever < 39	5,151	13.45	4,431	13.75	113	11.92	0.164
Headache	5,081	13.26	4,045	12.55	109	11.50	**0.024**
Hoarseness	175	0.46	203	0.63	12	1.27	**0.001**
Injection site reactions	4,713	12.30	4,063	12.61	119	12.55	**0.017**
Itchiness other than injection site	650	1.70	547	1.70	4	0.42	**0.009**
Nausea	2,038	5.32	1,571	4.88	50	5.27	**0.016**
Sleep disruption	487	1.27	463	1.44	17	1.79	**0.022**
Heartburn	560	1.46	469	1.46	12	1.27	0.997
Fatigue	7,028	18.35	6,256	19.41	164	17.30	**0.001**
Nervousness	7	0.02	16	0.05	0	0.00	0.057
Irritation	10	0.03	20	0.06	0	0.00	0.054
**Major side effects**							
Wheezing	51	0.13	77	0.24	3	0.32	**0.002**
Swelling of lips, face and\or throat	125	0.33	119	0.37	8	0.84	**0.005**
Loss of consciousness	90	0.23	47	0.15	2	0.21	**0.03**
Epileptic seizure	34	0.09	27	0.08	0	0.00	0.691
Other SE	5,838	15.24	5,186	16.09	200	21.10	**0.001**
Total	**38,309**		**32,223**		**948**		
All reported SE	**71,480**						

*p*-values in bold are significant at < 0.05.

### 3.3. Side-effects of COVID-19 vaccination by vaccine dosage

The reported AEs in relation to the doses were as follows: 53,910 (75.42%), 17,369 (24.30%) and 201 (0.28%) after receiving the first, second, and third vaccine doses, respectively (Table 3). The most common AEs reported after receiving the first dose were: fatigue 10,174 (18.87%), others 8,451(15.68%), fever < 39°C 7,395 (13.72%), headache 6,965 (12.92%), and injection site reactions 6,735 (12.49%). For the second dose, the most common AEs were fatigue 3,233(18.61%), others 2,745 (15.8%), fever < 39°C 2,271 (13.08%), headache 2,245(12.93%), and injection site reactions 2,143 (12.34%). For the third dose, the most common AEs were: fatigue 41 (20.4%), fever < 39°C 29 (14.43%), other AEs 28 (13.93%) and headache 25 (12.44%). The occurrences of severe AEs were reported as follows: 444 (0.82%), 138 (0.79%), and 1 (0.50%), after receiving the first, second, and third vaccine doses, respectively. Fever and epileptic seizure were the only statistically significant AEs amongst the number of vaccine doses received (*p*-value < 0.05), whereby the occurrence of these vaccine side-effects was higher in the first dose recipients, followed by the second and then third doses. However, seizure was a very rare occurrence and constituted only 0.08% of all reported AEs (Table 3).

**Table 3:** Adverse events in relation to the number of vaccine doses received.

	**Dose 1**		**Dose 2**		**Dose 3**		**P-value**

	***n***	**%**	***n***	**%**	***n***	**%**	
**Minor side effects**							
Anxiety	328	0.61	104	0.60	1	0.50	0.956
Shortness of breath	1,275	2.37	458	2.64	3	1.49	0.081
Dizziness	2,578	4.78	819	4.72	7	3.48	0.532
Fever above 39	4,044	7.50	1,456	8.38	16	7.96	**0.001**
Fever < 39	7,395	13.72	2,271	13.08	29	14.43	**0.035**
Headache	6,965	12.92	2,245	12.93	25	12.44	0.857
Hoarseness	306	0.57	81	0.47	3	1.49	0.062
Injection site reactions	6,735	12.49	2,143	12.34	17	8.46	0.068
Itchiness	92	0.17	34	0.20	0	0.00	0.66
Itchiness other than injection site	824	1.53	247	1.42	4	1.99	0.519
Nausea	2,742	5.09	900	5.18	17	8.46	0.119
Sleep disruption	743	1.38	221	1.27	3	1.49	0.549
Heartburn	770	1.43	265	1.53	6	2.99	0.157
Fatigue	10,174	18.87	3,233	18.61	41	20.40	0.526
Irritation	23	0.04	7	0.04	0	0.00	0.947
Nervousness	21	0.04	2	0.01	0	0.00	0.206
**Major side effects**							
Wheezing	107	0.20	24	0.14	0	0.00	0.218
Swelling of lips, face and\or throat	188	0.35	63	0.36	1	0.50	0.927
Loss of consciousness	94	0.17	45	0.26	0	0.00	0.073
Epileptic seizure	55	0.10	6	0.03	0	0.00	**0.027**
Other SE	8,451	15.68	2,745	15.80	28	13.93	0.473
**Total**	**53,910**		**17,369**		**201**		
All reported SE	**71,480**						

*p*-values in bold are significant at < 0.05.

### 3.4. Associations between demographic factors and COVID-19 vaccine side-effects

A logistic regression analysis was performed to explore the associations between AEs and the following variables: age, gender, vaccine dosage, and vaccine type. Interestingly, the majority of these significantly associated vaccine side-effects were minor. The following AEs were significantly associated with age: injection site pain (*P* < 0.001), injection site redness (*P* = 0.008), headache (*P* < 0.001), fatigue (*P* = 0.001), and sleep disturbances (*P* = 0.029). Whilst itching was significantly associated with age (*P* = 0.034), Pfizer-BioNTech (*P* < 0.001), and Oxford-AstraZeneca (*P* = 0.009), low fever was significantly associated with age (*P* < 0.001), gender (*P* = 0.019), and second vaccine dose (*P* = 0.039) (Table 4).

**Table 4:** Logistic regression analysis for associations between vaccine minor and/or major adverse events and demographic variables.

**Demographic variables**	**P-value**	**95% confidence interval**
**Age**		
Injection site pain	< 0.001	(0.9939, 0.9978)
Injection site redness	0.008	(0.9897, 0.9984)
Low fever	< 0.001	(0.9927, 0.9965)
Fever >39	< 0.001	(0.9911, 0.9956)
Headache	< 0.001	(0.9920, 0.9958)
Itching	0.034	(1.0011, 1.0285)
Fatigue	0.001	(0.9951, 0.9986)
Sleep disturbance	0.029	(0.9894, 0.9994)
**Gender (female)**		
Low fever	0.019	(1.010, 1.1162)
**Dose 2**		
Low fever	0.039	(0.8745,.9967)
**Pfizer-BioNTech**		
Fever >40	0.004	(1.149, 1.321)
Itching	< 0.001	(0.0375, 0.218)
**Oxford-AstraZeneca**		
Itching	0.009	(0.118, 0.734)

### 3.5. ICU admission post-COVID-19 vaccination

There were 10 ICU admissions post COVID-19 vaccination (five males and five females) with a mean age of 38.4 ± 13.8 years of age. All cases required mechanical ventilation. Three patients had comorbidities. The mean of days between vaccination and ICU admission was 8.2 ±3.8 days.

As shown in Table 5, five ICU cases were reported after receiving the first dose of Oxford-AstraZeneca. Two cases had COVID-19 pneumonia 8–14 days after vaccination. One case of acute myocardial infarction in a previously healthy 32 years old male 4 days following the first dose of Oxford-AstraZeneca. One case experienced acute respiratory failure 11 days after vaccine administration. One case of Guillain-Barré syndrome was reported in a previously healthy 39 years old male 10 days following his first dose of Pfizer-BioNTech vaccine. A case of septic shock was reported in a 60-year male with a coronary arterial disease, 5 days following the first dose of Pfizer-BioNTech. A case of grand mal seizure was reported 7 days following the second dose of Pfizer-BioNTech. One patient aged 28 years suffered from intracranial hemorrhage 12 days following her second dose of Pfizer-BioNTech (Table 5).

**Table 5:** ICU Admissions post COVID-19 vaccination.

**Case**	**Gender**	**Age**	**Vaccine**	**Dose**	**Days between vaccine and ICU admission**	**Pre-ICU reported side effects**	**ICU diagnosis**	**Comorbidities**
**Mean** ±**SD**		**38.4** ±**13.8**			**8.2** ±**3.8**			
1	M	32	Oxford-AstraZeneca	1	4	-	Acute myocardial infarction	-
2	M	32	Oxford-AstraZeneca	1	8	Dyspnea -Fever above 39 -Headache -Hoarseness -Injection site pain -Wheezing -Fatigue	Pneumonia\ COVID-19	-
3	F	34	Oxford-AstraZeneca	1	14	-Dizziness - Fever above 39 - Headache	Pneumonia\COVID-19	-
4	F	43	Oxford-AstraZeneca	1	10	Dyspnea	Atherosclerosis	-
5	M	51	Oxford-AstraZeneca	1	11	Low fever	Acute respiratory failure	Chronic lung disease
6	F	28	Pfizer-BioNTech	2	12	Headache	Intracranial hemorrhage	-
7	F	31	Pfizer-BioNTech	2	7	-	Grand mal seizures	Hypertension
8	M	39	Pfizer-BioNTech	1	10	-	Guillain-Barré syndrome	-
9	F	60	Pfizer-BioNTech	2	4	-	Abnormal blood gas levels	-
10	M	60	Pfizer-BioNTech	1	5	-Anxiety - Fever above 39	Septic shock	Chronic cardiovascular disease

## 4. Discussion

In this study, we examined the spectrum and occurrence of AEs in relation to COVID-19 vaccine types and number of doses. The majority of reported AEs were mild and were not associated with hospitalization. The COVID-19 pandemic has caused a global crisis on all fronts including health, society, and economy. To combat the pandemic that has impacted the daily lives of many people, the FDA has granted emergency use authorization for Pfizer-BioNTech, Oxford-AstraZeneca, and Moderna vaccines. Indeed, with a record that beats the fastest vaccine that had previously been developed and approved for mumps virus in the 1960's which took four years, this in turn has raised some concerns regarding the safety and efficacy of these COVID-19 vaccines and the potential adverse events (12, 15, 17). According to the Saudi MOH databases, up to 23.18 million individuals completed the initial COVID-19 vaccination protocol (two vaccine doses) and 2.7 million individuals completed the third vaccine “booster” doses during the study period. The findings from this retrospective nationwide study, with a sample size of 28,031 participants who self-reported their side-effects through digital governmental platforms, represent important insights at the times of modern medicine. Indeed, this study emphasizes the importance of patient-centered care and the employment of technology in telemedicine which can facilitate health-care providers and patients alike.

Notably, the results of this study align with the low occurrence of major AEs after COVID-19 vaccination (18–23). The reported AEs in relation to vaccine doses were 53,910 (75.42%), 17,369 (24.30%), and 201 (0.28%) after the first, second, and third vaccine dose, respectively. The low number of AEs reported following the third dose might be associated with a lower uptake for the third dose. However, the higher rate of reporting of AEs after the first dose may reflect the type of the vaccine administered or could be related to reporting bias as people may tend to be more motivated to report AEs initially with the increase in vaccine uptake and safety. In one study, the rate of AEs was lower with heterologous vaccination than homologous vaccination (24).

There had been no reported anaphylaxis after COVID-19 vaccines in this study. Shimabukuro et al. delineated that 21 cases of anaphylaxis were reported in the United States following the first dose of Pfizer-BioNTech vaccine in December 2020 with a rate of 11.1 cases per million vaccine doses administered (25). In a systematic review, the occurrence of anaphylactic reaction after Pfizer-BioNTech and Moderna vaccines was associated with an overall pooled prevalence estimate of 5.0 (95% CI 2.9 to 7.2, I2 = 81%, *p* = < 0.0001) (26). Furthermore, Cirillo et al. stated that various vaccine side-effects affecting the orofacial (mouth and face area) were reported after receiving both Pfizer-BioNTech and Oxford-AstraZeneca vaccines. The authors stated that these events were rare, which included acute peripheral facial paralysis (Bell's palsy), facial swelling, and swelling of the lips, face or tongue associated with anaphylaxis (27).

In our study, two of the major reasons for ICU admissions following COVID-19 vaccination were the development of acute myocardial infarction in one patient and Guillain-Barré in another patient who received AstraZeneca and Pfizer-BioNTech vaccines respectively. Indeed, the occurrence of myocardial infarction and acute myocarditis following COVID-19 vaccination had been reported in several case reports and case series following different types of COVID-19 vaccines (28–32). In a case-control study, the incidence of myocardial infarction and stroke was 6.18 vs. 5.49 per 1,000,000 person-days in those who were never vaccinated vs. fully vaccinated individuals (33). Another study showed no significant increased risk of myocardial infarction in the first 14 days following COVID-19 vaccine (34). The occurrence of Guillain-Barré syndrome after COVID-19 vaccination had been reported in case series and reports (35, 36). In a head-to-head comparison, the adjusted relative risk was 20.56 (95% CI, 6.94–64.66) in Ad.26.COV2.S vs. mRNA vaccines (36). One study showed that the occurrence of Guillain-Barré syndrome 1–21 days after COVID-19 vaccination with mRNA vaccines was 1.3 per 100,000 person-years. This rate was not different from the usual rate in the community (37). Finally, two ICU cases in our study developed COVID-19 pneumonia 8–14 days after vaccination. It is worth noting here that none of the vaccines administered were live-attenuated, and therefore, it would be medically implausible to assume that the vaccines induced COVID-19.

To the best of our knowledge, this is the first nationwide registry-based study in KSA that provided insights into the patterns and spectrum of COVID-19 vaccine side-effects derived from self-reported data. Indeed, the present study has constituted a relatively large sample size (>28,000 study participants) who reported vaccine side-effects through the Sehhaty mobile application. However, the bias of self-reporting could not be ruled out. In addition, the majority of the participants were apparently healthy as only a small number has reported comorbidities. This may bias the spectrum of reported AEs. Moreover, the data was based on the use of an online mobile application, and so the collected data may not be representative of the whole population in KSA, especially in regions with limited internet connection. Also, the reported vaccine side-effects were based on a limited checkbox selection questionnaire which was not verified by a healthcare professional.

In conclusion, our findings revealed that the most prevalent COVID-19 vaccine side-effects in Saudi Arabia were mild in their severity; adverse events to COVID-19 vaccines at any dose were extremely rare and only 0.03% required intensive care admission. Indeed, this information will help reduce vaccine hesitancy amongst the wider community and encourage further mass vaccination and continued advocacy for vaccination to combat and end the COVID-19 pandemic, especially as second booster doses are now available. Further studies are warranted to obtain a better understanding of the association between risk factors and the experiencing of side-effects post-vaccination.

## Data availability statement

The datasets presented in this article are not readily available because restrictions apply to the availability of these data, which were used under license for the current study, and so are not publicly available. Data are however available from the authors upon reasonable request and with permission from the Saudi MOH. Requests to access the datasets should be directed to the corresponding authors.

## Ethics statement

The studies involving human participants were reviewed and approved by the Institutional Review Board (IRB) committee from the General Directorate for Research and Studies (GDRS) (IRB No. 21-50M) at the Saudi Ministry of Health (MOH), Riyadh, Saudi Arabia. The Ethics Committee waived the requirement of written informed consent for participation.

## Author contributions

JMA and AA: conceptualization, data collection, design methodology and analysis, manuscript writing, and editing. JAA: conceptualization, manuscript writing, data analysis, and interpretation. BFA: data collection, analysis, methodology, and manuscript writing. AHA, NMA, MMA, and WS: data collection, analysis, and manuscript editing. TA and MHQ: data collection and interpretation. MB and MA: conceptualization, manuscript editing, methodology, and supervision. All authors reviewed and approved the final version of the manuscript.
